# Passionately demanding: Work passion’s role in the relationship between work demands and affective well-being at work

**DOI:** 10.3389/fpsyg.2023.1053455

**Published:** 2023-02-09

**Authors:** Catarina Cabrita, Ana Patrícia Duarte

**Affiliations:** ^1^Iscte Instituto Universitário de Lisboa, Lisbon, Portugal; ^2^Business Research Unit, Iscte Instituto Universitário de Lisboa, Lisbon, Portugal

**Keywords:** work demands, work passion, affective well-being at work, challenge stressors, hindrance stressors, harmonious passion, obsessive passion

## Abstract

In a world marked by exponential change, work demands are intensifying and becoming increasingly prominent in organizations’ reality. Work demands are stressors for the employees who must deal with these requests as they bring with them costs. Promoting these workers’ well-being at work is important as their level of comfort is closely related to how they will behave in the workplace. In this context, work passion is a fundamental factor in employees’ daily motivation to work well. This study tested a new approach to work demands, distinguishing between challenges and obstacles, exploring how they influence affective well-being at work when work passion is part of the equation. Individual workers also participate in how demands are formulated, which affects their level of well-being at the workplace. Data were collected with an online questionnaire administered to a sample of 515 participants who had been working in the same organization for at least 6 months. The results of multiple regression analysis show that the way demands are revealed influences what kind of work passion predominates and thus how much workers’ well-being at work is altered. Harmonious passion emerges as a personal resource that has the power to prevent negative affective states related to work from developing, while obsessive passion ends up putting even more demands on employees and having a stronger negative association with their affective well-being in the workplace.

## 1. Introduction

Workplaces are undergoing a profound transformation due to the challenges presented by the exponential growth in technologies’ use and globalization, as well as the resulting organizational restructuring and changes in workloads and employment contract typologies (Di Fabio, 2017). Heavy work demands are increasingly common because organizations need to respond to these trends. These additional demands can provide benefits, including the achievement of goals through improved performance and of short-term results due to the process intensification required to optimize workflow (LePine et al., 2005; Kubicek and Korunka, 2017). Positive psychology suggests that workers’ well-being at work is imperative so that these improvements’ positive consequences can be experienced by healthy workers who feel good in their workplace (Bakker and Demerouti, 2007; Oliveira et al., 2020). Well-being at work has also multiple consequences for individuals, such as physical and mental health issues and their satisfaction with life (Diener, 2012), therefore, should be nurtured by organizations. In fact, organizations have the obligation to safeguard workers’ psychological welfare (e.g., European Pact for Mental Health and Well-Being). The literature offers a wide variety of definitions and conceptualizations of well-being. It can be conceptualized from a context-free perspective as related to people’s feelings about their life in general (Diener and Ryan, 2009) or connected to specific parts of their life such as work (Brief, 2001; Warr, 2002). Well-being can be defined as “an optimal psychological experience that promotes (a high) functioning” workforce (Ryan and Deci, 2001, p. 142) and a stronger focus on job tasks. One important aspect is affective well-being, which is a cumulation of positive emotions related to different domains of life that can be transferred to work contexts. Therefore, in the current research focused on individuals’ affective well-being at work (Warr, 1990), that reflects a higher frequency of experiences producing positive affect (e.g., happy, comfortable) and an absence of experiences associated with negative affect (e.g., anxious, sad). In addition, work passion emerges as a motivational process capable of empowering individuals to respond effectively to the different types of demands they face. This passion reflects employees’ tendency to engage more fully in significant activities (Vallerand et al., 2003). Previous research has explored the relationship between work passion and well-being at work (Curran et al., 2015; Pollack et al., 2020), suggesting that a significant link between the constructs might exists.

The business world today is characterized by growing demands and constant challenges, so managers must understand how their staff’s passion for their work influences their well-being at work through the demands made on them. Thus, the present study sought to extend the existing understanding of how work demands are related to workers’ affective well-being at work. More specifically, this research examined work demands’ (i.e., on-the-job challenges or obstacles) influence on employees’ well-being at the workplace *via* the mediation of work passion (both harmonious and obsessive passion).

The next section offers an overview of the literature on work demands, well-being at work, and work passion that theoretically frames the research hypotheses and theoretical model proposed for the present study.

## 2. Literature review

### 2.1. Work demands

Work demands “refer to psychological, physical, social and organizational aspects that require physical or psychological skills and efforts and (that) are therefore associated with certain physiological and/or psychological costs” (Bakker and Demerouti, 2007, p. 312) that can end up being workplaces’ defining characteristic. Scholars initially asserted that work demands are only associated with negative factors and thus constitute psychological stressors, but, more recently, these demands have been shown to be associated with more than just lower levels of well-being (Kubicek and Korunka, 2017; Schaufeli, 2017).

One of the most significant models developed to analyze work demands and the effect they have on workers’ well-being is the job demands-resources (JD-R) approach (Bakker and Demerouti, 2007). This model postulates that workplaces’ characteristics have varied impacts on employees’ health and that these features can be divided into two categories: demands and resources. Demands are not automatically negative because they only become stressors when the effort required to respond to demands goes beyond what workers can handle (Bakker and Demerouti, 2007). Resources are necessary not only to mitigate work demands’ possible negative effects but also to contribute indirectly to a higher level of well-being in terms of personal development and growth.

This approach to work resources fits well with Hobfoll’s (2001) conservation of resources theory (COR), which states that humans’ main motivation is to maintain and accumulate resources as these become the means by which individuals can reach and protect other resources. One major criticism of the JD-R model is that it focuses too much on workplace characteristics and exclusively on demands and resources while neglecting the potential for each worker’s personal resources to improve their well-being. These assets are “psychological characteristics or aspects of the self that are generally associated with resilience and refer to the ability to successfully control and impact the environment” (Schaufeli and Taris, 2014, p. 49).

To reflect the evolving literature on this topic, LePine et al. (2005) proposed a two-dimensional view of work demands: challenges (e.g., work overload, work complexity, time pressure and responsibility) and obstacles (e.g., job insecurity, interpersonal conflict, bureaucracy and organizational politics, role conflict and role ambiguity). This model predicts different consequences according to workers’ exposure to the two components. Challenges are associated with greater job satisfaction, while obstacles are associated with lower job satisfaction (Boswell et al., 2004; Crawford et al., 2010).

The argument underlying this approach to work demands is that both challenges and obstacles are demanding on a personal level. More specifically, “challenges have the potential to promote mastery, personal growth and future earnings, while obstacles can prevent personal growth, learning and the achievement of goals” (Schaufeli and Taris, 2014, p. 52). Not unexpectedly, the two aspects of work demands have a positive relationship with burnout (Bakker and Demerouti, 2007; Wu et al., 2019), but the link between demands and workers’ involvement changes depending on the kind of demand. Challenges and involvement have a positive relationship, while obstacles and involvement have a negative connection. Resources are further negatively associated with burnout and positively related to involvement (Sawhney and Michel, 2021).

### 2.2. Work demands and well-being at work

The present study focused on affective well-being at work. Given that the emotional side is more intense and momentary, makes sense the approach of well-being as being a predominant emotional state (Daniels, 2000). The theoretical framework included Warr’s (1990) multidimensional approach to affective well-being at work based on two orthogonal dimensions related to work: pleasure and activation. Individuals’ well-being is measured as their positioning in these dimensions. The further away an affective state is from the central point, the more the state intensifies depending on its placement in one of four quadrants: anxiety (i.e., low pleasure and high activation), enthusiasm (i.e., high pleasure and high activation), depression (i.e., low pleasure and low activation), and comfort (i.e., high pleasure and low activation). From this perspective, challenges have recently been observed to enhance a positive work perspective and contribute to increased motivation. Obstacles, in turn, have been found to have a negative impact on motivation and thus an association with more intense burnout (Crawford et al., 2010; Van den Broeck et al., 2010; Mazzola and Disselhorst, 2019; Jiang et al., 2020; Rai and Thakur, 2020).

The current research, therefore, expected challenges would have a positive relationship with workers’ well-being at work while obstacles would have a negative link with individuals’ affective well-being at work. The following research hypothesis and two subhypotheses were defined based on the above findings:

*H1*: The relationship between work demands and affective well-being at work depends on the type of demand.

*H1a*: Challenges have a positive relationship with workers’ affective well-being.

*H1b*: Obstacles have a negative relationship with workers’ affective well-being.

### 2.3. Work passion as a mediator of work demands and well-being’s relationship

Work passion emerges as a motivational process that enables employees to respond to different types of demands. This passion is reflected in workers’ tendency to engage in significant activities that require them to expend energy, so they eventually internalize these behaviors as part of their own identity (Vallerand et al., 2003). Considering to the dualistic model of passion (Vallerand et al., 2003), two distinct types of passion have been identified: harmonious and obsessive.

The first type, i.e., harmonious passion, “results from an autonomous internalization of the activity … (as part of) the person’s identity” (Vallerand et al., 2003, p. 757). Harmonious passion refers to individuals’ voluntary acceptance of behaviors as important and relevant and promotes harmony with the rest of their life. As for obsessive passion, it occurs when “control (becomes part) of the internalization of the activity in the person’s identity” (Vallerand et al., 2003, p. 757). This obsession is often caused by intra-personal pressures and/or interpersonal factors related to feelings of self-esteem or social acceptance or by the uncontrollable level of arousal generated by the activities in question. Obsessive passion’s consequences are diverse. Obsessed individuals commonly manifest feelings of frustration when full involvement in the relevant behaviors is impossible and low levels of pleasure when performing activities in other areas of their life (Vallerand et al., 2003). These people also experience lower levels of happiness, job satisfaction, physical health, and well-being, which has repercussions for, among other things, their perception of self-efficacy and sense of belonging. These impacts contrast sharply with those of harmonious passion (Curran et al., 2015; Moè, 2016; Gong et al., 2018; Schellenberg et al., 2018).

The two types of passion’s different impacts on individuals means that passion interacts in varied ways with job demands. The latter are tasks that require some effort and that are associated with specific costs (Bakker and Demerouti, 2007), so these activities can potentially control workers, creating discomfort, malaise, and exhaustion much like obsessive passion does (Vallerand et al., 2003). Demands can feed employees’ motivations through obsessive passion and force them to deal with their job tasks in rigid and inadequate ways, which produces lower levels of well-being (Trépanier et al., 2013).

In contrast, resources facilitate greater involvement in work as staff members have enough resources available to deal with the existing pressures. These individuals internalize the relevant activities voluntarily as part of their identity, which generates meaning and a feeling of fulfillment in their work without creating conflicts with other parts of their life. Resources not only contribute to employees’ greater involvement and increased well-being but also have the power to prevent burnout through harmonious passion (Trépanier et al., 2013; Yukhymenko-Lescroart and Sharma, 2022).

Researchers’ results change when work demands are examined as challenges or obstacles. Challenges have been associated with greater involvement in job tasks, while obstacles are more strongly associated with greater burnout (Crawford et al., 2010; Van den Broeck et al., 2010; Mazzola and Disselhorst, 2019; Jiang et al., 2020). These demands’ influence on employees’ well-being varies depending on the typology applied (Crane and Searle, 2016). According to Van den Broeck et al. (2010), “obstacles are threatening restrictions, which drain energy and provoke an overcoming (strategy) focused on emotion … (while) challenges are obstacles that can be overcome and that require energy, but are simultaneously stimulating” (p. 741). Harmonious passion, in turn, generates a fuller involvement that can facilitate concentration and foster positive affect, but obsessive passion creates an internal feeling of compulsion that forces the relevant individuals to get involved in the required activities.

Hence, work passion can be proposed as a possible mediating mechanism between work demands and affective well-being at work. Work passion is strongly linked with individual motivation. The self-determination theory (Ryan and Deci, 2001) distinguishes two forms of motivation - intrinsic motivation and extrinsic motivation, and both drive and energize human behavior. Motivation is always about energy, direction, persistence, and scope of a final result that can be obtained in different ways (Ryan and Deci, 2001). As “passion can nourish motivation, increase well-being and give meaning to everyday life” (Vallerand et al., 2003, p.756), it can be considered as a driving force, complementary to motivation. Human beings end up finding certain activities that are preferable to others due to the level of pleasure and satisfaction that is obtained in their performance, but also because the activities contribute to defining who each person is by “regularly engaging in passionate activities that provide a recurring dose of happiness, there will be a profound impact on people’s psychological functioning” (Vallerand, 2012, p.47). In short, the passion determines the preference for the target, while motivation drives the person to trace a journey to the target, for this reason, passion always has the motivational process adjacent (Vallerand, 2015).

Given the above findings, the present study’s second hypothesis and its two subhypotheses were formulated as follows:

*H2*: Passion for work mediates the relationship between work demands and affective well-being at work.

*H2a*: Harmonious passion mediates the relationship between challenges and affective well-being.

*H2b*: Obsessive passion mediates the relationship between obstacles and well-being.

However, a different approach is needed to reflect how obstacles have the power to impede personal growth and the achievement of goals (Schaufeli and Taris, 2014). Conservation of resources theory (Hobfoll, 2001) states that individuals have cognitive and environmental resources that protect them from the stressors generated by work demands, which has repercussions for individuals’ well-being (e.g., Stahl et al., 2018; Muhamad et al., 2020). Resources are thus valued and are multiplicative as they can be accumulated (i.e., gain spirals) or depleted (i.e., loss spirals; Hobfoll, 2001).

Gain spirals enhance a positive process of growth and resilience that produces less wear and tear in the long term (Schaufeli et al., 2009) as harmonious passion is linked with a positive relationship with work based on greater involvement. In gain spirals, the connection employees establish with their work is capable of equipping them to acquire the resources needed to deal with work demands (Birkeland et al., 2017). The current research thus included an additional subhypothesis that proposed the following:

*H2c*: Harmonious passion mediates the relationship between obstacles and affective well-being.

The nature of obsessive passion further requires a new approach to the expected relationship between challenges and well-being at work. As mentioned previously, this type of passion presupposes workers’ involvement in activities in rigid ways (Vallerand, 2015), even when challenges allow these individuals to grow, engage in constant learning (Schaufeli and Taris, 2014), and experience a higher level of well-being (e.g., Boswell et al., 2004; Crawford et al., 2010; Crane and Searle, 2016). These positive outcomes may themselves eventually control employees as benefits can increase the intra-or interpersonal pressures to meet work demands (Philippe et al., 2009; Vallerand, 2015). The reason why challenges can foster obsessive passion is that, regardless of how activities are internalized, humans are driven to reach their fullest potential and satisfy their psychological needs (Ryan and Deci, 2017). Challenges ultimately enable individuals to surmount obstacles and experience self-realization and growth, thereby integrating challenges into workers’ identity (Vallerand et al., 2003).

However, obsessive passion has also been associated with a lack of satisfaction of needs at work when other aspects of life fail to compensate for the absence of fulfillment (Vallerand, 2015; Pollack et al., 2020). These workers tend to continue to engage in frustrating activities and end up losing control over these behaviors (Lalande et al., 2015). The final subhypotheses proposed for the present study was, therefore, as follows:

*H2d*: Obsessive passion mediates the relationship between challenges and affective well-being.

**Figure 1:**
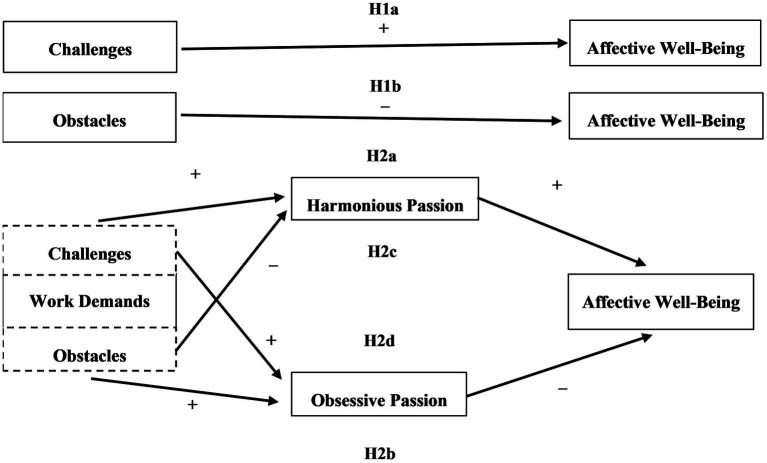
Research model. H, Hypothesis.

Figure 1 presents the resulting research model.

## 3. Materials and methods

### 3.1. Procedures and participants

To test the research model using empirical methods, a quantitative, correlational, and cross-sectional study was conducted based on data gathered with an online questionnaire distributed to workers from different organizations. The procedures followed the ethical research guidelines of Portugal’s Order of Psychologists and the Declaration of Helsinki. The questionnaire was created using Qualtrics Survey software and shared in social networks (e.g., LinkedIn and Facebook). This instrument included a home page with information about the study’s aims, an informed consent form emphasizing that participation was voluntary and that the data were confidential, and instructions designed to diminish evaluation apprehension (Podsakoff et al., 2003). To take part in the present research, the workers had to have been employed by their current organization for at least 6 months. This inclusion criterion assured that the participants were sufficiently familiar with their workplace’s characteristics. The average response time expected was 10 min.

The final sample of 515 workers was obtained after eliminating incomplete surveys and screening for the inclusion criterion. Slightly more than half of the participants were females (50.7%). Their ages ranged from 20 to 68 years old [mean = 42.50; standard deviation (SD) = 9.47]. Nearly half had a higher education degree (47.6%), 32.4% had completed secondary education, 14.6% had a master’s degree, 4.3% had up to 9 years of schooling, and 1.2% had a doctorate. The participants had worked in their organization for a maximum of 43 years (mean = 14.56; SD = 10.22; minimum = 6 month). The majority were not in management (68.5%), and almost all worked full-time (97.7%). More than four-fifths of the respondents had a permanent employment contract (84.3%).

The data were collected during a national lockdown period with imposed teleworking due to the coronavirus disease-19 pandemic, so the questionnaire asked the participants about their current work regime. Nearly two-thirds reported that they were fulfilling their professional duties in the usual workplace (62.5%), but 27.8% were teleworking and 9.7% were following a hybrid work regime.

Regarding the organizations’ sector of activity, the secondary sector was the most predominant (i.e., 258 participants or 50.1%). In addition, 77.9% worked for a private company. Finally, 60.8% of the respondents worked for a large organization with 250 workers or more.

### 3.2. Measures

#### 3.2.1. Affective well-being at work as a criterion variable

This construct was measured using the Institute of Work Psychology Multi-Affect Indicator Scale developed by Warr (1990). The scale’s 12 items describe positive and negative affective states varying according to individuals’ level of activation (e.g., “unhappy” or “motivated”). The response scale allowed the participants to report how often their work made them experienced that state in the previous few weeks (i.e., 1 = “Never”; 6 = “All the time”). This instrument had previously been validated for the Portuguese population by Gonçalves and Neves (2011).

However, the literature shows some divergence in how well-being at work should be measured. Some authors assert that this variable needs to be studied as one construct, while other researchers emphasize the importance of dividing well-being into positive and negative indicators (Warr, 2012). Various recent studies have measured affective well-being (e.g., Zito et al., 2019) with an indicator based on positive feelings and another on negative ones. The present investigation opted for an overall affective well-being at work score as, according to Warr (2012), “positive and negative factors may, in practice, be artifacts arising from response acquiescence and other biases rather than a genuine conceptual bifurcation and it may be preferable to examine affect scores with alternative content combined” (p. 3). The measure used in the current research presented a Cronbach’s alpha (*α*) of 0.79.

#### 3.2.2. Work demands as a predictor variable

This variable was measured using the work stressor scale developed by Lepine et al. (2016). This instrument comprises 20 items in which half correspond to challenges (e.g., “I have a high level of responsibility”; *α* = 0.87) and the other half to obstacles (e.g., “I have conflicts with colleagues”; *α* = 0.85). The response scale ranged from 1 (“Never”) to 5 (“Very often”) in order to assess the recurrence of specific types of work demands in the participants’ daily on-the-job activities.

#### 3.2.3. Work passion as a mediator

The work passion scale developed by Vallerand and Houlfort (2003) was used to assess work passion. This instrument was validated for the Portuguese population by Martins et al. (2014). The scale consists of 14 items in which 7 measure harmonious passion (e.g., “My work gives me access to a variety of experiences”; *α* = 0.90) and the other 7 assess obsessive passion (e.g., “My mood depends on my ability to do my job”; *α* = 0.87). The response scale ranged from 1 (“Strongly disagree”) to 7 (“Strongly agree”).

#### 3.2.4. Control variables

Based on the literature reviewed for this study, control variables were included in the survey, namely, if the participants had any work exemptions (Brito, 2016), had been previously promoted in their present organization, and/or expected to be promoted within a year (Astakhova and Porter, 2015). These items were answered dichotomously (0 = “no”; 1 = “yes”).

### 3.3. Common method variance

The current research’s data were gathered from a single source at a single time, so Common method variance’s (CMV) possible occurrence was a concern (Podsakoff et al., 2012; Bozionelos and Simmering, 2022). The present survey included a marker variable to address this issue. The literature reviewed indicated that organizational hypocrisy has not previously been shown to be related on a theoretical level to the other constructs in this study, which made organizational hypocrisy appropriate as a marker variable (Lindell and Whitney, 2001). This construct can be defined as the belief that an organization claims to be something it is not (Wagner et al., 2009). Wagner et al. (2009) created a six-item scale to measure workers’ perception of organizational hypocrisy, so this instrument was incorporated into the current survey but with the procedure developed by Babu et al. (2019) to help workers evaluate their organization’s perceived hypocrisy (e.g., “My organization keeps its promises”). The seven items were evaluated using a response scale ranging from 1 (“Strongly disagree”) to 5 (“Strongly agree”).

The data analysis revealed that organizational hypocrisy presents statistically significant moderate correlations with all the variables of interest. Ideally, no significant correlations should exist, or, if they do, they need to be quite weak (Lindell and Whitney, 2001). The present results showed that the correlations were quite significant, so the marker variable technique failed to exclude the possibility of CMV. This variable was instead included as a covariate in the hypothesis testing phase.

To address the issue of CMV further, Harman’s single-factor test was applied because it facilitates CMV’s identification. This technique postulates that, if significant bias is present, it will either originate from a single variable in the factor analysis or a general construct will account for most of the covariance between all the measures (Podsakoff et al., 2012). In the present study, a single factor was not identified as the source of bias, and the most predominant construct accounts for only 23.6% of the 68.03% total variance explained (Kaiser-Meyer-Olkin test = 0.91; Bartlett’s test = 16444.24; *p* < 0.001). Therefore, any CMV in the sample is not significant enough to produce bias.

## 4. Results

### 4.1. Descriptive statistics and correlations

Table 1 lists the means, SDs, and Spearman’s correlations. The variables of interest are not all significantly correlated as challenges are not related with harmonious passion and well-being at work and obstacles are not correlated with obsessive passion. The sociodemographic variables of gender, tenure in organization, and work regime at the time of the survey are, however, significantly correlated with affective well-being, so they were included in subsequent analyses as covariates.

**Table 1:** Means (Ms), standard deviations (SDs), Spearman’s correlations, and Cronbach’s alpha (*α*) values.

	*M*	SD	1	2	3	4	5	6	7	8	9	10	11	12
1. Gender	–	–												
2. Tenure in organization	14.56	10.22	−0.41**											
3. Working conditions	–	–	0.40**	−0.24**										
4. Schedule exemption	–	–	−0.007	−0.04	0.11**									
5. Past promotion	–	–	−0.23**	0.44**	−0.03	0.06								
6. Expectation of future promotion	–	–	−0.02	−0.06	0.04	−0.003	0.17**							
7. Organizational Hypocrisy (Marker)	3.45	1.36	0.001	0.004	−0.02	−0.04	−0.12**	−0.22**	(0.91)					
8. Challenge stressors	3.74	0.61	0.08	0.03	0.12**	0.06	0.07	0.03	0.12**	(0.87)				
9. Obstacles stressors	2.40	0.65	−0.06	0.14**	0.000	−0.02	−0.06	−0.12**	0.47**	0.36**	(0.85)			
10. Harmonious passion	5.02	1.23	−0.03	0.12**	0.01	0.05	0.18**	0.24**	−0.40**	0.083	−0.24**	(0.90)		
11. Obsessive passion	3.31	1.31	0.02	0.11*	−0.03	0.14**	−0.16**	−0.09*	−0.22**	0.16**	−0.03	0.48**	(0.87)	
12. Affective well-being at work	3.77	0.62	−0.24**	0.11*	−0.12**	0.02	0.11*	0.19**	−0.42**	−0.07	−0.38**	0.51**	0.13**	(0.79)

Number = 515; Gender: 0 = male, 1 = female; Working conditions: 1 = usual place, 2 = hybrid regime, 3 = telework; Schedule exemption: 0 = no, 1 = yes; Past promotion: 0 = no, 1 = yes; Expectation of future promotion: 0 = no, 1 = yes; internal consistency coefficients measured by Cronbach’s *α* given in parenthesis; *p < 0.05, **p < 0.01.

### 4.2. Hypotheses testing

Hayes (2018) reports that a mediation model can be worth analyzing even when a statistically significant relationship between *X* and *Y* is absent. The current research used Model 4 of Hayes’ Process Macro to conduct simple mediation analysis. The residues’ homogeneity and normality were checked, as well as the variance inflation factor values (below 2.85) and tolerance values (above 0.35). The results thus indicate that multicollinearity is absent (Daoud, 2017).

The variables treated as covariates (i.e., gender, seniority in organization, working conditions, organizational hypocrisy, schedule exemption, past promotion, and expectation of future promotion) were included in all the analyses to avoid possible bias. Each analysis also incorporated work demands as the predictor variable as a covariate because challenges and obstacles were significantly correlated.

The first group of hypotheses posited that the relationship between work demands and affective well-being at work differed depending on the type of demand. The analysis first focused on challenges’ total effect on affective well-being, showing that the impact is positive but not significant [beta (*B*) = 0.06; bootstrap confidence interval (CI) of 95% = (−0.02; 0.14)]. Despite going in the expected direction, this relationship is not statistically significant, so H1a was not supported by the data. Obstacles’ total effect is, in contrast, significant and negative as was expected [*B* = −0.27; bootstrap CI of 95% = (−0.35; −0.18)], so H1b received support from the data. That is, the more present obstacles are at work, the lower workers’ levels of affective well-being become.

The second set of hypotheses focused on work passion’s mediation of the relationship between work demands and affective well-being at work. H2a proposed that harmonious passion mediates the relationship between challenges and affective well-being. The results reveal that challenges’ effect on harmonious passion is both positive and significant [*B* = 0.32; bootstrap CI of 95% = (0.15; 0.48)]. Namely, the more significant challenges are in the workplace, the higher employees’ levels of harmonious passion becomes. Harmonious passion’s effect on affective well-being is equally positive and significant [*B* = 0.22; bootstrap CI of 95% = (0.18; 0.26)], so, the higher the level of harmonious passion is, the stronger workers’ affective well-being becomes at work.

Challenges’ indirect impact on well-being at work *via* harmonious passion is also positive and significant [*B* = 0.07; bootstrap CI of 95% = (0.03; 0.11)]. This finding indicates that challenges’ presence increases workers’ harmonious passion for work, which then contributes to increased well-being. H2a was thus supported by the data. In addition, harmonious passion has a full mediation effect since challenges’ direct impact on well-being is not significant [*B* = 0.02; bootstrap CI of 95% = (−0.06; 0.09)] and the total effect is also insignificant. The relationship between challenges and affective well-being is evidently completely indirect and entirely contingent on an increase in harmonious passion.

H2b proposed that obsessive passion mediates the relationship between obstacles and affective well-being at work. The results indicate that obstacles’ impact on obsessive passion is negative and not statistically significant [*B* = −0.01; bootstrap CI of 95% = (−0.21; 0.20)], so whether fewer or more obstacles exist at work does not appear to affect the level of obsessive passion. In contrast, obsessive passion’s effect on affective well-being is negative and significant [*B* = −0.07; bootstrap CI of 95% = (−0.10; −0.03)], which means that the greater workers’ obsessive passion is for their job, the lower their well-being becomes. The indirect effect is positive but nonsignificant [*B* = 0.00; boot CI of 95% = (−0.02; 0.02)], so H2b was not verified.

H2c posited that harmonious passion mediates the relationship between obstacles and affective well-being at work. Obstacles’ impact on harmonious passion is negative and significant [*B* = −0.30; bootstrap CI of 95% = (−0.48; −0.12)], indicating that the presence of obstacles reduces employees’ harmonious passion for their work. Harmonious passion’s effect on well-being is, in turn, positive and significant [*B* = 0.22; bootstrap CI of 95% = (0.18; 0.26)], which means that stronger harmonious passion is associated with more affective well-being at work. The indirect impact is negative and significant [*B* = −0.07; bootstrap CI of 95% = (−0.11; −0.02)], which suggests that, when obstacles are present at work and harmonious passion acts as a mediator, obstacles have a less negative effect on affective well-being. However, the mediation effect is only partial since obstacles’ direct effect on well-being is statistically significant [*B* = −0.20; bootstrap CI of 95% = (−0.28; −0.12)]. Overall, harmonious passion mediates the relationship between obstacles and affective well-being and helps attenuate the intensity of the variables’ negative relationship. In other words, H2c was supported by the data.

Finally, H2d proposed that obsessive passion mediates the relationship between challenges and affective well-being at work. The results confirm that challenges’ impact on obsessive passion is positive and significant [*B* = 0.38; bootstrap CI of 95% = (0.19; 0.57)], so, the more challenges dominate the workplace, the stronger workers’ obsessive passion becomes. In addition, this passion’s effect on affective well-being is negative and significant [*B* = −0.07; bootstrap CI of 95% = (−0.10; −0.03)], indicating that higher levels of obsessive passion are linked with lower affective well-being. The indirect impact is negative and significant [*B* = −0.03; bootstrap CI of 95% = (−0.05; −0.01)], which shows that, when challenges are present at work and their effect is mediated by obsessive passion, challenges have a negative effect on affective well-being. Given that challenges’ direct impact is nonsignificant [*B* = 0.02; bootstrap CI of 95% = (−0.06; 0.09)], obsessive passion’s mediation is complete. These findings confirm that H2d is valid.

**Figure 2:**
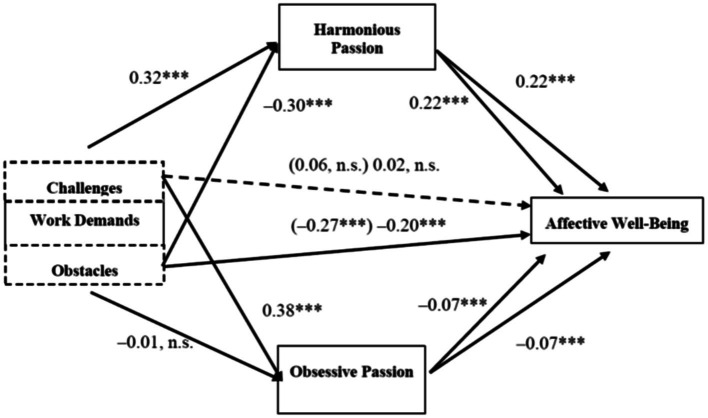
Main findings. Non-standardized coefficients; total effects reported in parentheses; n.s., non-significant; ***p < 0.001.

A significant mediation model was obtained that explains 41.8% of affective well-being at work’s unique variance [coefficient of determination (*R^2^*) = 0.42; *F-*statistic (*F*)*_(11,503)_* = 32.88; statistical probability (*p*) < 0.001]. This explained variance is larger than the variation explained by work demands (*R^2^* = 0.30; *F_(9,505)_* = 23.83; *p* < 0.001), which means that work passion contributes significantly to clarifying this relationship. The covariates included did not have a significant impact on well-being at work, with the exception of gender [*B* = −0.24; bootstrap CI of 95% = (−0.34; −0.14)] as women reported overall lower well-being at work. Figure 2 presents the study’s main results.

## 5. Discussion

The present research sought to contribute to a fuller understanding of how work demands are connected to workers’ affective well-being at work and work passion’s role in this relationship. The conceptual model reflected a dual perspective on work demands—distinguishing between challenges and obstacles—and on work passion—differentiating between harmonious and obsessive passion. This approach facilitated analyses of the different ways that work demands can influence workers’ affective well-being on the job, incorporating work passion as a buffer or amplifier of work demands’ impacts on employees’ well-being.

The findings reveal that the link between challenges and affective well-being at work is positive but not strong enough to be statistically significant, so H1a received insufficient support. However, researchers have recently found evidence that challenges are increasingly associated with positive emotions that translate into greater personal fulfillment and more positive results for workers. Challenges also contribute to a stronger motivation to work among employees who believe that their efforts in response to challenges will ultimately be valued by their organization (Rai and Thakur, 2020). The direction of affective well-being and challenges’ relationship can be more easily understood as a change in perspective, in which challenges’ role in promoting workers’ growth is much stronger than the distress and discomfort generated by challenges’ associated costs.

That this relationship is not statistically significant could be due to this study’s wider context. With the pandemic, the participants’ work regimes were often different from their normal working conditions (i.e., hybrid and remote). In addition, changes had been made regarding, among other things, communication, performance assessment, work-family balance, number of working hours, and organization of job tasks (International Labor Organization, 2020), which may have altered the kinds of challenges workers face. The present research’s division of work demands into challenges and obstacles was based on the existing literature, so this approach could be overly simplistic (Li et al., 2019) for the complex transition period during which the data were collected. Future studies need to assess the relationship between challenges and affective well-being further to evaluate its stability in other contexts. Analyses of individuals’ subjective assessments of what is personally more or less challenging (Zigarmi et al., 2009) may also provide a better understanding of this connection.

The link between obstacles and affective well-being at work is negative and statistically significant. Self-determination theory (Ryan and Deci, 2017) suggests that people naturally seek to make the most of their abilities and talents in order to achieve optimal levels of psychological functioning and well-being. Obstacles make reaching the maximum potential difficult for individuals, which results in a lower level of well-being. H1b was thus supported by the current results. This relationship’s direction was as expected as the associated subhypothesis postulated that workers must make a special effort and deal with the costs linked with the specific skills needed to respond to demands.

These findings confirm the results reported by other researchers (Bakker and Demerouti, 2007; Crawford et al., 2010; Van den Broeck et al., 2010; Mazzola and Disselhorst, 2019; Jiang et al., 2020). Further attention still needs to be paid to the direction of demands’ relationship with workers’ affective well-being at work, and, as previously discussed, other studies have demonstrated demands’ negative and harmful effects on employees’ health and affective well-being (Sonnentag et al., 2010; Stahl et al., 2018). The present results thus provide support for the idea that high demands produce short-term stress and cause workers to experience lower well-being over time (Muhamad et al., 2020). However, other variables (e.g., work regime) may also have influenced the current findings.

The current results for work passion’s role are quite interesting. The hypotheses postulated that harmonious passion mediates the relationship between challenges and affective well-being at work (H2a). Obsessive passion should also increase when more obstacles are present, and this type of passion is associated with lower levels of well-being (H2b). In addition, harmonious passion mediates the link between obstacles and affective well-being (H2c), and obsessive passion mediates the relationship between challenges and affective well-being (H2d).

The data provide support for H2a, so, the more often challenges recur, the more workers will be praised for their harmonious passion and thus the higher their level of affective well-being at work will be. The critical points raised by the JD-R model (Bakker and Demerouti, 2007) include that personal resources allow employees to meet their professional goals and achieve personal development goals, which is also a fundamental feature of challenges (Schaufeli and Taris, 2014). These benefits can combine with work passion’s incorporation of professional activities into individuals’ self-image, thereby contributing to employees’ identity (Vallerand et al., 2003; Forest et al., 2011), stimulating greater engagement, and increasing their work resources (Trépanier et al., 2013). The above links suggest that harmonious passion not only diminishes negative affective states but also builds up personal resources when workers have control over their on-the-job activities, maintain a balance between work and other domains of their life, and experience personal and professional development.

Harmonious passion’s effects also fit well with the premises of self-determination theory (Ryan and Deci, 2017). This finding is significant because passion has been conceptualized as a potential motivational process that allows individuals to respond in positive ways to the demands put on them at work (Vallerand et al., 2003). Strong passion functions as a resource and source of inspiration that increase employees’ level of well-being when they have to deal with challenging work demands, which confirms the beneficial effects detected in prior studies of harmonious passion (Vallerand et al., 2007; Trépanier et al., 2013; Gong et al., 2018; Schellenberg et al., 2018).

Next, H2b predicted increased obsessive passion when obstacles arise more frequently and, as a result, lower affective well-being at work, but the present research did not confirm this subhypothesis. H2b was developed for various reasons. First, evidence has been found for a positive relationship between demands, obsessive passion, and burnout (Trépanier et al., 2013). Second, researchers have observed that work demands are associated with specific costs (Bakker and Demerouti, 2007) and that obstacles can hinder personal development and goals’ achievement (Schaufeli and Taris, 2014). Individuals seek to reach their maximum potential to experience satisfaction through competence, autonomy, and helpful relationships (Ryan and Deci, 2017). However, workers may be motivated by obsessive passion to deal with work demands in rigid, inappropriate ways that ultimately undermine their health and well-being (Trépanier et al., 2013).

Obsessive passion’s mediation was not confirmed for the present sample, but this type of passion’s significant negative effect on affective well-being at work needs cannot be ignored given obsessive passion’s adverse impact on the well-being of workers who tend to be more obsessive (e.g., Vallerand et al., 2010; Trépanier et al., 2013). One possible explanation for the absence of a statistically significant relationship between obstacles and obsessive passion could be that obstacles have the power to “prevent personal growth, learning and the achievement of goals” (Schaufeli and Taris, 2014, p. 52). Obstacles can thus become stressors associated with less motivation to react appropriately to obstacles since the effort expended primarily depletes employees’ resources and produces ineffective responses. Even if the results are positive, they may still not be gratifying for workers (Rai and Thakur, 2020). Obsessive passion may lead to internalization of activities, yet this process inevitably require individuals to expend time and energy on finding solutions. Passion is always associated with motivations (Vallerand, 2015), so obstacles’ relationship with affective well-being appears completely antagonistic due to obstacles’ negative impact on workers’ motivation (Rai and Thakur, 2020).

The current research provided support for H2c, namely, a significant negative relationship between obstacles and affective well-being at work when this link is mediated by harmonious passion. These results indicate that, the more demands prevent personal growth and goals’ achievement (Schaufeli and Taris, 2014), the less harmonious passion employees will experience since obstacles generate tension and anxiety and thus potentially decrease these individuals’ enthusiasm and motivation (Wood and Michaelides, 2015). Obstacles also prevent workers from doing what they love (Wilson and Britt, 2020). However, harmonious passion’s effect on affective well-being under these conditions remains positive and significant even though this relationship is evidently weaker than challenges’ connection with well-being.

The present findings underline passion’s importance as a motivational mechanism, in which employees’ internalization of on-the-job activities allows these individuals to filter out obstacles’ negative affective consequences and strengthen their personal sense of affective well-being at work. In this context, resource conservation theory (Hobfoll, 2001) suggests that individuals contain within themselves the resources to deal with the stressors generated by work demands (e.g., Halbesleben, 2006). These assets are exceptionally valuable because they can help workers reach other goals as well.

From this perspective, resources are multiplicative as they can be accumulated (i.e., gain spirals) or depleted (i.e., loss spirals; Hobfoll, 2001). Gain spirals are guided by a positive process of resilience and growth associated with less wear and tear in the long term (Schaufeli et al., 2009). Harmonious passion is associated with a positive relationship with work, so the assumption can be made that this kind of passion generates a gain spiral because the constructive connection established with job tasks can ensure workers have the necessary resources to deal with demands (Birkeland et al., 2017). However, the current results suggest that obstacles weaken harmonious passion, which in turn has harmful consequences that reduce affective well-being. Obstacles cause employees to use up their resources, thereby ultimately diminishing these individuals’ harmonious passion for their work because obstacles can impede growth and generate entropy in personal development (Rai and Thakur, 2020) and may even force workers to leave their organization as a last resort (Abbas and Raja, 2019).

The current results also confirm that a significant positive relationship exists between challenges and obsessive passion, which then has a significant negative link with affective well-being at work. These connections imply that people who predominantly feel obsessive passion at work tend to experience lower levels of well-being. The findings thus provide support for obsessive passion’s mediation of the relationship between challenging demands and affective well-being (i.e., H2d). When workplace activities control employees, they experience negative emotions if they become less frequently involved in those job tasks (Vallerand et al., 2003), which explains the significant impact of obsessive passion’s indirect link with well-being as a mediator of obstacles and challenges’ effect on affective well-being.

The present study thus confirmed the most basic assumption made about obsessive passion: the activity controls the person (Vallerand et al., 2003; Philippe et al., 2009). Obsessive passion has been associated with workers’ dissatisfaction regarding how well their needs are met in the workplace. Even the demands that can contribute to these individuals’ growth end up trapping them because they will obsessively engage in activities and eventually lose control over their behavior (Lalande et al., 2015; Vallerand, 2015).

In addition, self-determination theory (Ryan and Deci, 2017) assumes that individuals will remain focused on reaching their maximum potential, which brings up the following issue. People are currently facing challenging demands, and yet individuals are driven to reach their fullest potential, grow personally, and move toward additional future achievements (Schaufeli and Taris, 2014). The question arises of whether workers could be going through a phase in which challenges can generate obsessive passion because employees’ idea of what constitutes success is defined by outside sources. In this case, obsessively passionate workers would tend to see work demands as an inconvenience (Lavigne et al., 2014), and even challenges would have a negative effect when mediated by this demotivating process.

The above line of reasoning can be combined with the theory of conservation of resources (Hobfoll, 2001) and a focus on loss spirals to describe how stressors contribute to an on-going loss of resources—or access to them—and lead to greater burnout (Weigl et al., 2010). Loss spirals can increase obsessive passion’s impact on affective well-being as workers cannot disconnect from thoughts about their job activities, frequently feel frustrated, and derive less pleasure from their undertakings in other areas of their life (Vallerand et al., 2003). All these propensities contribute to diminishing resources because work demands tend to be seen as obstacles by individuals driven by internal pressures to engage in work activities (Lavigne et al., 2014; Amarnani et al., 2019).

### 5.1. Limitations and future lines of research

The present research had limitations that need to be considered when interpreting its results and that can be addressed by further investigations. Correlational studies are restricted in terms of ascertaining the causal relationships between the relevant variables. This investigation’s cross-sectional nature also increased the probability of CMV being present (Bozionelos and Simmering, 2022). The inclusion of a marker variable failed to rule out this problem, so Harman’s single-factor test was selected as an alternative way to check for bias. However, the latter technique has been criticized as insensitive and limiting (Podsakoff et al., 2003). A longitudinal design could thus strengthen the current findings and mitigate this limitation in future research. In addition, the sample was gathered using a convenience sampling strategy that limits the results’ generalizations to other contexts. Further investigations of this topic may benefit from collecting more representative samples of the relevant population.

Qualitative information could also be gathered on work passion. Given the similarity between this construct and motivation, researchers may gain interesting insights into workers’ perceptions of the difference between these two variables, especially if individuals from different age groups are included due to their developmental stages and associated needs. To enrich future studies’ findings or extend the results, new variables could be included to maximize the present proposed research model’s explanatory power, namely, subjective evaluations of what type of passion employees feel. Recent investigations have discovered that passion is a dynamic process that originates from workers’ incessant need to make sense of—and to be able to interpret changes in—their work experiences (Egan et al., 2019). Thus, scholars also need to focus on how individuals carry out cognitive assessments of their working conditions and on-the-job experiences rather than just how they evaluate the presence of stressors.

### 5.2. Theoretical and practical implications

The current research contributed to consolidating the theoretical model most often used to analyze work demands’ impacts on workers by highlighting the role played by personal resources such as work passion. In addition, a closer look at this passion provided a deeper understanding of other motivational mechanisms as recent studies have tended to focus on psychological states that drive humans to act as they do (e.g., engagement and workaholism) and the corresponding results (i.e., higher or lower levels of well-being and health). This empirical research specifically concentrated on work passion’s role as an antecedent of psychological states that motivate individuals to respond to demands in positive ways, that is, employees’ predisposition to integrate tasks into their identity and their overall intensity’s effect on subsequent behavior.

On a practical level, management can benefit from a fuller understanding of work demands and the distinct impact they have on workers’ affective well-being at work, including gaining deeper insights into how employees’ identity and well-being are initially conditioned by their company. To increase their workers’ affective well-being at work, organizations should reduce the number of obstacles in the workplace (e.g., role conflict and role ambiguity) while offering resources that help workers deal with multiple demands in their jobs (e.g., work overload). While challenges appear to be less prejudicial than obstacles, the former comprise demands that organizations need to manage and/or avoid. Harmonious passion for work must be nurtured as it can be an important resource that helps employees cope with heavy work demands and experience greater well-being at work. Work-family balance practices can further promote workers’ harmony in the rest of their life, in conjunction with efforts to avoid an overwork climate.

Organizations’ attention to these issues will ensure employees can cope better with work stressors and will mitigate the latter’s negative effect on affective well-being. The extant literature highlights workplace well-being as the key to a set of desirable outcomes including, among others, good performance, organizational citizenship behavior, retention, and creativity at work. Organizations have an ethical obligation to safeguard workers’ psychological welfare (e.g., European Pact for Mental Health and Well-Being). However, making this an official policy is also a matter of strategic interest because of affective well-being at work’s implications in terms of employees’ attitudinal, emotional, and behavioral responses inside and outside the workplace.

## 6. Conclusion

Work passion has been found to provide individual and organizational advantages (Zigarmi et al., 2009). The demands put on employees are growing exponentially, so determining this passion’s potential impact on workers’ well-being at work has become of paramount importance as the way that each person internalizes their on-the-job activities will dictate the way they deal with these duties. Work passion’s interaction with demands also indicates that how organizations present their needs to employees has an impact on their workplace identity. Overall, these factors’ multifaceted nature shows that organizational life is a two-way street. Workers affect their organization, and their organization has an effect on them. The issue at hand is not merely about passion or organizational goals but instead about identity and people management. Work passion takes many forms, while demands involve meeting many standards. Ultimately, passionately demanding organizations play a key role in employees’ affective well-being at work.

## Data availability statement

The raw data supporting the conclusions of this article will be made available by the authors, without undue reservation.

## Ethics statement

Ethical review and approval was not required for the study on human participants in accordance with the local legislation and institutional requirements. The patients/participants provided their written informed consent to participate in this study.

## Author contributions

CC and AD: conceptualization, methodology, formal analysis, writing–original draft preparation, and writing–review and editing. CC: data collection. AD: project supervision. All authors have read and agreed to the published version of the manuscript.

## Funding

This research was partially supported by Portugal’s Fundação para a Ciência e Tecnologia (Grant UIDB/00315/2020 and contract DL 57/2016/CP1359/CT0004).

## Conflict of interest

The authors declare that the research was conducted in the absence of any commercial or financial relationships that could be construed as a potential conflict of interest.

## Publisher’s note

All claims expressed in this article are solely those of the authors and do not necessarily represent those of their affiliated organizations, or those of the publisher, the editors and the reviewers. Any product that may be evaluated in this article, or claim that may be made by its manufacturer, is not guaranteed or endorsed by the publisher.
