# Corrigendum: Passionately demanding: work passion's role in the relationship between work demands and affective well-being at work

**DOI:** 10.3389/fpsyg.2023.1233821

**Published:** 2023-06-23

**Authors:** Catarina Cabrita, Ana Patrícia Duarte

**Affiliations:** ^1^Iscte Instituto Universitário de Lisboa, Lisbon, Portugal; ^2^Business Research Unit, Iscte Instituto Universitário de Lisboa, Lisbon, Portugal

**Keywords:** work demands, work passion, affective well-being at work, challenge stressors, hindrance stressors, harmonious passion, obsessive passion

In the published article, there was an error in the Funding statement as published. The grant number was provided as “UID/03125/2020” but should be “UIDB/00315/2020”. The correct Funding statement appears below.

